# Gestational diabetes mellitus diagnosed at 24 to 28 weeks of gestation in older and obese Women: Is it too late?

**DOI:** 10.1371/journal.pone.0225955

**Published:** 2019-12-16

**Authors:** Wonjin Kim, Soo Kyung Park, Yoo Lee Kim

**Affiliations:** 1 Department of Internal Medicine, Division of Endocrinology and Metabolism, CHA Gangnam Medical Center, CHA University School of Medicine, Seoul, Korea; 2 Yonsei University College of Medicine, Seoul, Korea; 3 Departmentof Epidemiology and Biostatistics, School of Public Health, University of Maryland, College Park, Maryland, United States of America; East Tennessee State University, UNITED STATES

## Abstract

**Aim/Background:**

The prevalence of elderly pregnancy and maternal obesity is increasing worldwide. In old and obese women, metabolic derangement affecting fetal growth might be present earlier than the diagnosis of gestational diabetes mellitus (GDM) or even before pregnancy. We thus investigated whether GDM diagnosed at 24–28 weeks of gestation had already affected fetal abdominal growth and, if so, whether elderly pregnancy and/or maternal obesity aggravate fetal abdominal obesity.

**Methods:**

We retrospectively reviewed the medical records of 7820 singleton pregnant women who had been universally screened using a 50-g glucose challenge test (GCT) at 24–28 weeks of gestation, and underwent a 3-h 100-g oral glucose tolerance test (OGTT) if GCT were ≥140mg/dl. GDM and normal glucose tolerance (NGT) were diagnosed using the Carpenter-Coustan criteria. Fetal abdominal obesity was investigated by assessing the fetal abdominal overgrowth ratios (FAORs) of the ultrasonographically estimated gestational age (GA) of abdominal circumference per actual GA by the last menstruation period, biparietal diameter or femur length, respectively. Fetal abdominal overgrowth was defined as FAOR ≥ 90^th^ percentile. The subjects were divided into four study groups: group 1 (age < 35 years and pre-pregnancy body mass index [BMI] < 25 kg/m^2^), group 2 (age < 35 years and ≥ 25), group 3 (age ≥ 35 years and BMI < 25), and group 4 (age ≥ 35 years and ≥ 25).

**Results:**

The overall prevalence of GDM was 5.1%, with old and obese group 4 exhibiting the highest prevalence (22.4%). FAORs were significantly higher in the fetus of those with GDM than in the NGT subjects. But, in the subgroup analysis, only old and nonobese group 3 and old and obese group 4 with GDM exhibited significantly higher FAORs than the NGT subjects. Also, risk of fetal abdominal overgrowth was increased in group 3 and 4 subjects with GDM but not in young and nonobese group 1 GDM. The risk of fetal abdominal overgrowth significantly increased with maternal age >35 years, pre-pregnancy BMI >20kg/m^2^, and HbA1c >37.7 mmol/mol (5.6%). In multivariate analyses, maternal age and HbA1c were significantly associated with FAORs.

**Conclusion:**

GDM diagnosed at 24–28 weeks of gestation already affected fetal abdominal obesity in older and/or obese women, but not in younger and nonobese women. Our data suggest that selective screening and appropriate intervention of GDM earlier than 24–28 weeks of gestation might be necessary for high-risk old and/or obese women.

## Introduction

Gestational diabetes mellitus (GDM), defined as any degree of glucose intolerance with onset or first recognition during pregnancy, is associated with increased short- and long-term adverse outcomes for both the mother and the fetus [1]. The prevalence of GDM is increasing as a consequence of the increased prevalence of obesity and type 2 diabetes mellitus (DM) in addition to advancing maternal age [1,2].While there is much debate about the optimal GDM screening strategies, including universal versus selective screening and optimal timing for screening [3], many professional societies now recommend the universal GDM screening of pregnant women at 24–28 weeks of gestation [1,4–6]. This timeframe coincides with that of increased insulin resistance and allows sufficient time for treatment benefits. However, a high prevalence of GDM in early pregnancy (<20 weeks of gestation) has been reported in high-risk populations, such as obese and older women [3], and poor pregnancy outcomes were reported despite appropriate treatment [7]. These findings thus warrant early screening for GDM at least in these high-risk populations.

With the ongoing epidemic of obesity and diabetes resulting in a higher incidence of more type 2 DM in young women [8–10], and the likelihood that undiagnosed type 2 DM or prediabetes antedates the pregnancy, most guidelines recommend that screening for overt diabetes be performed during the initial prenatal visit, especially in high-risk groups [1,6,11]. But, the same glycemic threshold for diagnosing overt DM in the nonpregnant population is recommended in early pregnancy, and there is no recommendation in those guidelines for managing those with less severe hyperglycemia.

The International Association of Diabetes and Pregnancy Study Group (IADPSG) recommended that fasting plasma glucose (FPG) ≥92 mg/dL and <126 mg/dL in early pregnancy should be diagnosed as GDM [12] based on the results of the Hyperglycemia and Adverse Pregnancy Outcome (HAPO) study that associations between maternal glycemia and adverse outcomes are continuous across the range of glucose concentrations below the levels at which diabetes is diagnosed [13]. However, this recommendation is controversial because there is insufficient evidence to recommend the diagnosis and treatment of GDM before 24 weeks of gestation [5,14].

In Korea, all pregnant women are screened using a 50-g glucose challenge test (GCT) at 24–28 weeks of gestation and are diagnosed with GDM according to the results of the 100-g oral glucose tolerance test (OGTT). Also, most of the women undergo the glucose test during their initial prenatal visit and exclude overt diabetes [15]; however, in early pregnancy, the management strategy for women with glucose levels in the prediabetes range is not clearly established.

Increased fetal size, defined as macrosomia (≥4kg) caused by increased adiposity, is an important fetal complication of GDM that can be reduced by 50% with appropriate treatment [16,17]. The association between GDM and increased fetal adiposity is currently limited to late pregnancy [18–21] and birth [22–24]. However, emerging evidence indicates that abnormal fetal growth can occur at 20 weeks of gestation, well before the routine biochemical diagnosis of GDM at 24–28 weeks of gestation [25,26]. Although these findings call for an earlier diagnostic and intervention strategy for GDM to prevent abnormal fetal growth, the specific high-risk group requiring earlier screening and appropriate treatment remains to be identified.

In the present study, we aimed to determine whether fetal overgrowth and abdominal obesity were already present at the time of GDM diagnosis at 24–28 weeks of gestation and, if so, whether the known high-risk groups for the development of early GDM, namely, older and/or obese women, demonstrated a higher risk of fetal overgrowth and abdominal obesity.

## Materials and methods

### Subjects and data collection

We retrospectively reviewed the medical records of 7820 singleton pregnant women who were followed up at the outpatient clinic of CHA Gangnam Medical Center from January 1, 2012, to December 31, 2014. The data on maternal height, pre-pregnancy body weight, current body weight and GA at the 50-g glucose challenge test (GCT), biochemical test, and fetal biometry were obtained from the medical records. The data collection was approved by the Institutional Review Board of CHA Gangnam Medical Center with a waiver of informed consent for the retrospective chart review (IRB No.GCI-18-10).

### Diagnosis of GDM

**Fig 1:**
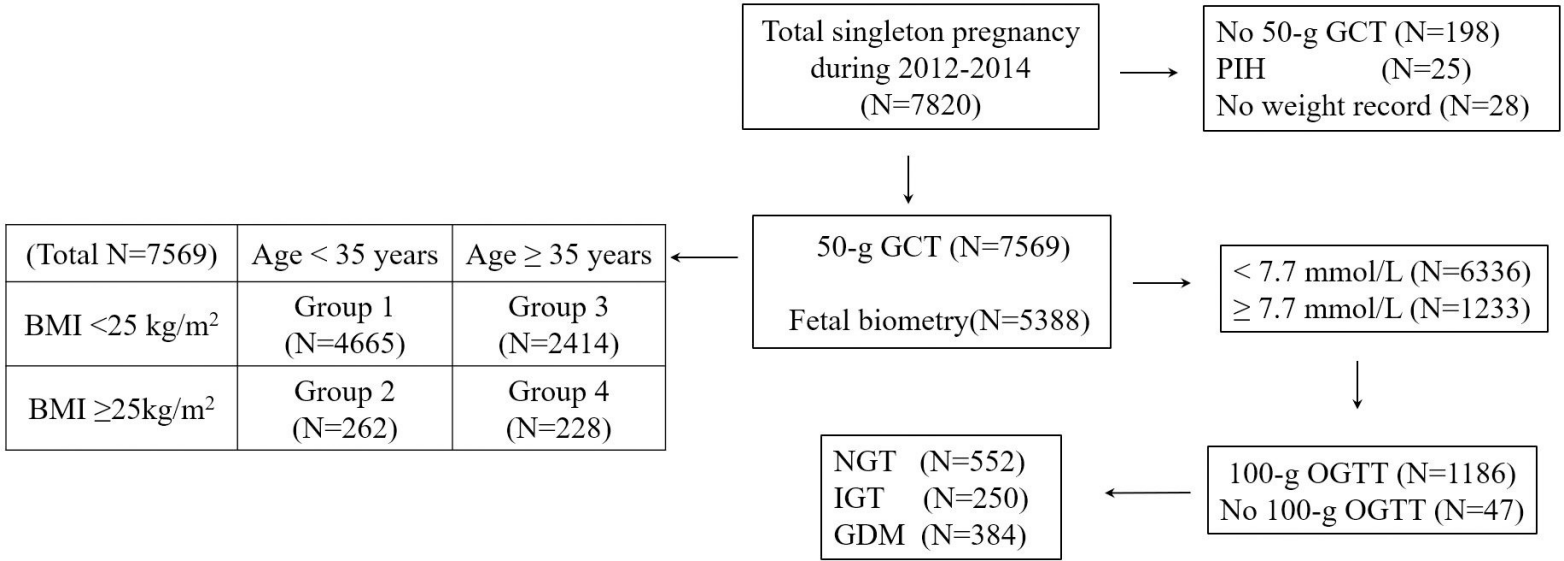
Study flow diagram. GCT, glucose challenge test; PIH, pregnancy induced hypertension; BMI, body mass index; NGT, normal glucose tolerance; IGT, impaired glucose tolerance; GDM, gestational diabetes mellitus, OGTT, oral glucose tolerance test.

All of the pregnant women were universally recommended to undergo screening with a 50-g GCT irrespective of fasting at 24–28 weeks of gestation and at 3-h 100-g OGTT with measurements of fasting plasma insulin and HbA1c after more than a 9-h fasting if the GCT result was ≥140 mg/dL. The diagnosis of GDM and normal glucose tolerance (NGT) depended on the Carpenter-Coustan criteria. Among the total group of 7820 subjects, 198 subjects without the results of a 50-g GCT, 25 subjects diagnosed with pregnancy induced hypertension before 24 weeks of gestation, and 28 women without pre-pregnancy body weight records were excluded from the study. A total of 7569 subjects screened with a 50-g GCT were included in the analysis. Of the 1233 women with ≥140 mg/dl glucose on the 50-g GCT, 47 did not undergo a 100-g OGTT, whereas 1186 (96.2%) did. Of these, 552 had NGT, 250 had impaired glucose tolerance, and 384 had GDM (Fig 1).

### Fetal biometry

Fetal biometry was measured simultaneously with the 50-g GCT in 5,388 pregnant women, and gestational dating was confirmed in 87% of these women by fetal ultrasonography performed prior to 14 weeks of gestation. Biparietal diameter (BPD), femur length (FL), and abdominal circumference (AC) were measured three times *via* ultrasonography (GE Healthcare, USA) by one of the experienced 3 sonographers, and the mean values were converted to each estimated GA (i.e., GA-BPD, GA-FL, and GA-AC) according to the Japanese fetal growth chart [27,28] (S1 Fig). But interobserver variability was not evaluated due to the retrospective nature of this study. We calculated fetal abdominal overgrowth ratios as GA-AC/GA-GCT (actual GA measured by the last menstruation period at the time of 50-g GCT and fetal biometry) to correct for the variations in the ultrasound scan timing, and GA-AC/GA-BPD or GA-AC/GA-FL to detect overgrowth of the abdomen relative to the head and femur growth, respectively. The presence of fetal abdominal overgrowth was defined as fetal abdominal overgrowth ratios ≥90^th^ percentile of the total subjects with fetal biometry (GA-AC/GA-GCT ≥ 1.08, GA-AC/GA-BPD ≥ 1.07, and GA-AC/GA-FL ≥ 1.07, respectively). The estimated fetal weight was calculated using the Shinozuka formula [29].

### Subgroup analysis

A total of 7569 subjects were divided into four study groups according to maternal age and pre-pregnancy BMI—group 1 (age < 35years and BMI < 25 kg/m^2^ [n = 4,665]), group 2 (age < 35 years and BMI ≥ 25 [n = 262]), group 3 (age ≥ 35 years and BMI < 25 [n = 2,414]), and group 4 (age ≥ 35 years and BMI ≥ 25 [n = 228])—and the prevalence of GDM was investigated in each group. We compared the clinical and biochemical parameters of mothers and fetal biometry data between 384 GDM and 6888 NGT subjects (6336 had <140 mg/dl glucose on the 50-g GCT, and 552 were normal on the 100-g OGTT). The GDM subjects in each group were named as groups 1, 2, 3, and 4 GDM. The GDM subgroup data were compared with each other and, also with those of the NGT subjects. For the calculation of the GDM prevalence in each group, women with impaired glucose tolerance on the 100-g OGTT were included in the NGT group; but, for the other comparisons women with impaired glucose tolerance were excluded from the NGT group.

### Biochemical analysis

Plasma glucose was measured using the hexokinase method (Quailigentglu, Sekisui, Japan), and HbA1c was measured *via* high-performance liquid chromatography (G8 Elution Buffer, Tosoh, Tokyo, Japan). The plasma insulin concentration was determined *via* electrochemiluminescence immunoassay (Elecsys Insulin, Roche Diagnostics GmbH, Mannheim, Germany). Insulin resistance (homeostatic model assessment for insulin resistance [HOMA-IR]) and secretion (HOMA-β) were calculated by homeostasis model assessment [30].

### Statistical analyses

All continuous variables were normally distributed, as determined by using the Kolmogorov-Smirnov test, and are expressed as mean ± SD values. Two data groups were compared by using an unpaired Student’s t-test. Four separate data groups were compared using analysis of variance (ANOVA) with Tukey’s method. Categorical data were compared using Fisher’s exact test. Correlations were assessed by using Spearman’s rank correlation coefficients (rho). To compare the clinical characteristics and fetal biometry of GDM in the four groups, categorized by the subjects’ age and pre-pregnancy BMI, we used ANOVA with Tukey’s method for multiple comparisons of paired groups. An unpaired Student’s t-test was used to compare the outcomes between the NGT group and one of the GDM subgroups. We conducted the logistic regression analysis or exact logistic regression analysis for the odds ratios (ORs) with 95% confidence intervals (CIs) to relate fetal abdominal overgrowth to maternal age at pregnancy, pre-pregnancy BMI, HbA1c, and GDM by subgroup. Finally, we examined the association between fetal abdominal overgrowth and age, BMI at the 50-g GCT, HbA1c, and HOMA-β by fitting multiple linear regression models. The significance level was set at 0.05. All of the analyses were performed using SAS 9.4 (SAS Institute, Cary, NC, USA).

## Results

### Prevalence of GDM according to maternal age and BMI

While the overall prevalence of GDM was 5.1% (384 of 7569) in this study, the prevalence of GDM significantly differed according to maternal age and BMI, with the lowest prevalence of 3.2% (147 of 4665) in the younger (<35 years) and nonobese (BMI <25 kg/m^2^) group 1, and the higher prevalence of 8.8% (23 of 262) and 6.8% (163 of 2414) in the younger and obese group 2 and older and nonobese group 3, respectively. The highest prevalence of 22.4% (51 of 228) was in the older and obese group 4 (S1 Table).

### Clinical and biochemical characteristics of the NGT and GDM subjects

The clinical and biochemical characteristics of the study subjects are presented in Table 1. Maternal age, pre-pregnancy BMI, BMI at the 50-g GCT, plasma glucose values on the 50-g GCT and 100-g OGTT, HbA1c, fasting plasma insulin, and HOMA-IR in the overall group of the GDM subjects were significantly higher than those of the NGT subjects, but the weight gain until the 50-g GCT and HOMA-β were not significantly different between the two groups (Table 1).

**Table 1:** Clinical and biochemical characteristics in the NGT and GDM subjects.

	NGT (n = 6888)	Total GDM (n = 384)	GDM			
			Group 1 (n = 147)	Group 2 (n = 23)	Group 3 (n = 163)	Group 4 (n = 51)
Age (years)	33.1 ± 3.8	35.3 ± 4.0^a^	31.7 ± 1.7 ^a^	31.2 ± 2.2	38.0 ± 2.6^a^^,^^b^^,^^c^	38.6 ± 2.6^a^^,^^b^^,^^c^
Pre-pregnancy BMI (kg/m^2^)	20.6 ± 2.6	22.3 ± 3.5 ^a^	20.6 ± 2.0	28.0 ± 2.8^a^^,^^b^	21.3 ± 2.0^a^^,^^c^	28.0 ± 2.4^a^^,^^b^^,^^d^
Weight gain ^e^ (kg)	7.6 ± 3.2	7.8 ± 3.6	8.3 ± 3.7	6.7 ± 4.3 ^a^	8.0 ± 3.4	6.9 ± 3.4 ^a^
50-g GCT (mmol/L)	6.2 ± 1.2	9.2 ± 1.4^a^	9.0 ± 1.1^a^	9.6 ± 1.4^a^	9.1 ± 1.1^a^	9.6 ± 2.4 ^a^
Fasting plasma glucose^f^ (mmol/L)	4.5 ± 0.4 (n = 552)	5.0 ± 0.8^a^	4.8 ± 0.6^a^	5.5 ± 0.9^a^^,^^b^	4.9 ± 0.6^a^^,^^c^	5.3 ± 1.5^a^^,^^b^^,^^d^
HbA1c (%) (mmol/mol)	5.0 ± 0.3 (31.3 ± 3.5) (n = 552)	5.3 ± 0.5^a^ (34.4 ± 6.0)	5.2 ± 0.3^a^ (33.4 ± 4.1)	5.6 ± 0.6^a^^,^ ^b^ (37.6 ± 6.9)	5.2 ± 0.3^a^^,^^c^ (33.9 ± 4.1)	5.6 ± 1.0^a^^,^^b^^,^^d^ (38.6 ± 10.8)
Fasting insulin^f^ (pmol/L)	54.9 ± 29.2 (n = 552)	68.8 ± 34.7^a^	65.3 ± 34.8	111.8 ± 35.4^a^^,^^b^	60.4 ± 26.4^c^	88.2 ± 32.6^a^ ^–^ ^d^
HOMA-IR	1.6 ± 0.9 (n = 552)	2.2 ± 1.3^a^	2.1 ± 1.2^a^	3.9 ± 1.4^a^^,^^b^	1.9 ± 0.9^c^	2.9 ± 1.2^a^ ^-^ ^d^
HOMA- β	183.9±109.8 (n = 552)	164.2 ± 117.6	166.9 ± 115.3	220.0 ± 145.4	140.5 ± 75.8^c^	176.6 ± 91.0

NGT, normal glucose tolerance; GDM, gestational diabetes mellitus; BMI, Body Mass Index; GCT, glucose challenge test; HbA1c, glycated hemoglobin; HOMA-IR, homeostatic model assessment for insulin resistance; HOMA-β, homeostatic model assessment for insulin secretion.

^a^p < 0.05, compared with the NGT subjects

^b^p<0.05, compared with group 1

^c^p < 0.05, compared with group 2

^d^p < 0.05, compared with group 3

^e^weight gain on the 50-g GCT from pre-pregnancy

^f^ on the 100-g OGTT

The HbA1c levels of all GDM subgroups were significantly higher than those of the NGT group. In particular, the HbA1c levels were significantly higher in groups 2 and 4 GDM than those in groups 1 and 3 GDM, respectively (37.6 ± 6.9 and 38.6 ± 10.8 vs. 33.4 ± 4.1 and 33.9 ± 4.1 mmol/mol, respectively; p < 0.0001). The HOMA-IR levels of all GDM subgroups except those of group 3 were significantly higher than those of the NGT group, and the obese groups 2 and 4 GDM exhibited higher values than the nonobese groups 1 and 3 GDM (3.9 ± 1.4 and 2.9 ± 1.2 vs. 2.1 ± 1.2 and 1.9 ± 0.9, respectively; p < 0.0001). The HOMA-β was not different between the GDM subgroups and the NGT group, but group 3 exhibited a significantly lower value than group 2 (140.5 ± 75.8 vs. 220.0 ± 145.4; p < 0.005) (Table 1 and S2 Fig).

### Fetal biometry of NGT and GDM the subjects

While the estimated fetal weight, GA-BPD, and GA-FL were not significantly different between the fetuses of the NGT and GDM subjects (Table 2), the GA-AC and all fetal abdominal overgrowth ratios of GA-AC/GA-GCT, GA-AC/GA-FL, and GA-AC/GA-BPD were significantly higher in the total GDM group than those in the NGT group. At the time of GDM screening and diagnosis, the fetal abdominal growth of the GDM subjects was already more advanced than that of the NGT subjects, even though the head and femur did not yet show signs of accelerated growth (Table 2).

**Table 2:** Results of fetal biometry in the NGT and GDM subjects.

	NGT (n = 5097)	Total GDM (n = 291)	GDM			
			Group 1 (n = 107)	Group 2 (n = 16)	Group 3 (n = 130)	Group 4 (n = 38)
GA-GCT (weeks)	26.4 ± 1.0	26.3 ± 0.9	26.4 ± 0.8	26.0 ± 0.8	26.3 ± 0.9	26.1 ± 1.3
GA-BPD (weeks)	26.9 ± 1.6	26.9 ± 1.5	27.0 ± 1.3	26.2 ± 1.6 ^a^	26.9 ± 1.4	26.8 ± 1.8
A-AC (weeks)	27.1 ± 1.4	27.4 ± 1.6 ^a^	27.3 ± 1.4	27.0 ± 2.1	27.5 ± 1.4 ^a^	27.4 ± 2.0
GA-FL (weeks)	26.9 ± 1.4	26.9 ± 1.3	27.0 ± 1.3	26.7 ± 1.5	26.9 ± 1.2	26.5 ± 1.6
EFW (gm)	1020 ± 164	1032 ± 168	1026 ± 144	971 ± 204	1040 ± 154	1032 ± 220
**Fetal Abdominal Overgrowth Ratio**						
GA-AC/GA-GCT^b^	1.03 ± 0.04	1.04 ± 0.05 ^a^	1.03 ± 0.05	1.04 ± 0.05	1.05 ± 0.04^a^	1.05 ± 0.04 ^a^
GA-AC/GA-FL	1.01 ± 0.04	1.02 ± 0.05 ^a^	1.01 ± 0.05	1.01 ± 0.04	1.02 ± 0.05 ^a^	1.03 ± 0.04 ^a^
GA-AC/GA-BPD	1.01 ± 0.05	1.02 ± 0.05 ^a^	1.01 ± 0.05	1.03 ± 0.05	1.02 ± 0.04 ^a^	1.03 ± 0.04 ^a^

NGT, normal glucose tolerance; GDM, gestational diabetes mellitus; GCT, glucose challenge test; HbA1c, glycated hemoglobin; GA-GCT, actual gestational age by last menstruation period (LMP) on the 50-g GCT and fetal biometry; GA-BPD, estimated gestational age by biparietal diameter; GA-AC, estimated gestational age by abdominal circumference; GA-FL, estimated gestational age by femur length; EFW, estimated fetal weight

^a^p < 0.05, compared with NGT subjects

^b^Actual gestational age by LMP on the 50-g GCT and fetal biometry

In the subgroup analysis, the fetal abdominal overgrowth ratios of the GA-AC/GA-GCT (1.05 ± 0.04 and 1.05 ± 0.04 vs. 1.03 ± 0.04; p <0.001), GA-AC/GA-BPD (1.02 ± 0.04 and 1.03 ± 0.04 vs. 1.01 ± 0.05; p <0.05), and GA-AC/GA-FL (1.02 ± 0.05 and 1.03 ± 0.04 vs. 1.01 ± 0.04; p <0.001) were significantly higher in the old groups 3 and 4 GDM than in the NGT subjects, but not in groups 1 and 2 GDM (Table 2). These results revealed that fetal abdominal growth was accelerated only in older and/or obese GDM subjects, but not in younger and/or obese GDM subjects.

In contrast with the well-known association of fetal abdominal overgrowth with maternal hyperglycemia and HbA1c level, fetal abdominal overgrowth was observed in the older and nonobese group 3 GDM with a lower mean FPG level of 4.9 mmol/L and HbA1c of 33.9 mmol/mol (5.3%) but not in the younger and nonobese group 1 GDM who had comparable FPG and HbA1c levels of 4.8 mmol/L and 33.4 mmol/mol (5.2%), respectively. The younger and obese group 2 with a higher FPG level of 5.5 mmol/L and HbA1c of 37.6 mmol/mol (5.6%) did not exhibit significant fetal abdominal overgrowth, even though the older and obese group 4 with comparable FPG and HbA1c levels of 5.3 mmol/L and 38.6 mmol/mol (5.7%), respectively, exhibited marked fetal abdominal overgrowth (Tables 1 and 2).

### Factors related to fetal abdominal overgrowth ratios

To identify the factors associated with fetal abdominal overgrowth ratios, correlation coefficients were calculated for all subjects. HbA1c exhibited the highest correlation with all three ratios (r = 0.1619, r = 0.1452, and r = 0.1537, respectively; p <0.0001; S2 Table). In addition, FPG on 100-g OGTT, BMI at the 50-g GCT, maternal age, pre-pregnancy BMI, and weight gain until the 50-g GCT exhibited significant positive correlations with these ratios, whereas HOMA-β exhibited a significant negative correlation. HOMA-IR did not exhibit a significant correlation with these ratios (S2 Table). However, for the GDM subjects, pre-pregnancy BMI did not exhibit a significant correlation, whereas HbA1c, FPG on 100-g OGTT, maternal age, BMI on the 50-g GCT, and weight gain on the 50-g GCT exhibited significant correlations with the fetal abdominal overgrowth ratios of GA-AC/GA-GCT and GA-AC/GA-FL (S3 Table). In all of the correlation analyses of the fetal abdominal overgrowth ratios, the BMI on the 50-g GCT exhibited a higher correlation coefficient than pre-pregnancy BMI.

### Risk of fetal abdominal overgrowth

The risk of fetal abdominal overgrowth, indicated by a fetal abdominal overgrowth ratio ≥ 90^th^ percentile of GA-AC/GA-GCT >1.08, significantly increased with maternal age above 35 years, pre-pregnancy BMI >20 kg/m^2^, and HbA1c >38 mmol/mol (5.6%) (Table 3). In addition, the risk was 2.15-fold in all the GDM subjects and increased to 2.63-fold in group 3 GDM and to 3.39-fold in group 4 GDM compared with the NGT subjects. However, the risk of fetal abdominal overgrowth was not significantly higher in the younger age groups 1 and 2 GDM than in the NGT subjects. Also, only groups 3 and group 4 GDM exhibited a significantly increased risk of abdominal overgrowth according to fetal abdominal overgrowth ratios of GA-AC/GA-BPD >1.07 and GA-AC/GA-FL >1.07 (Table 4).

**Table 3:** Risk of fetal abdominal overgrowth according to maternal age, pre-pregnancy BMI, and HbA1c.

Variables	Risk of Fetal Abdominal Overgrowth Ratio^#^ ≥ 90th Percentile Odds ratio (95% CI)			
Maternal age (years)	<30 (ref.)	30–35	35–40	>40
	1.00	1.25 (0.94, 1.67)	1.76* (1.30, 2.37)	1.94* (1.30, 2.90)
Pre-pregnancy BMI (kg/m^2^)	<20 (ref.)	20–25	25–30	>30
	1.00	1.55* (1.29, 1.87)	1.49* (1.03, 2.18)	2.90* (1.47, 5.70)
HbA1c (%)	<5.0 (ref.)	5.0–5.3	5.3–5.6	>5.6
	1.00	1.05 (0.62, 1.80)	1.58 (0.89, 2.81)	2.62* (1.31, 5.25)

BMI, body mass index; HbA1c, glycated hemoglobin

*p<0.05

^**#**^GA-AC/GA-GCT > 1.08 (GA-AC, estimated gestational age by abdominal circumference; GA-GCT, actual gestational age by last menstruation period [LMP] at 50-g GCT and fetal biometry)

**Table 4:** Risk of fetal abdominal overgrowth in total and subgroups of the GDM subjects.

	Risk of Fetal Abdominal Overgrowth Odds Ratio (95% CI)				
	Total GDM	GDM Group 1	GDM Group 2	GDM Group 3	GDM Group 4
GA-AC/GA-GCT ≥1.08	2.15* (1.57, 2.94)	1.11 (0.59, 2.09)	3.45 (0.80, 11.71)	2.63* (1.72, 4.04)	3.39* (1.64, 7.03)
GA-AC/GA-BPD ≥1.07	1.79* (1.29, 2.49)	1.68 (0.98, 2.89)	3.40 (0.79, 11.53)	1.82* (1.13, 2.93)	1.46 (0.57, 3.77)
GA-AC/GA-FL ≥1.07	1.65* (1.18, 2.32)	0.99 (0.51, 1.91)	0.67 (0.02, 4.42)	1.93* (1.21, 3.08)	3.35* (1.62, 6.94)

GDM, gestational diabetes mellitus; GA-AC, estimated gestational age by abdominal circumference; GA-GCT, actual gestational age by last menstruation period (LMP) on the 50-g GCT and fetal biometry; GA-BPD, estimated gestational age by biparietal diameter; GA-FL, estimated gestational age by femur length

*p<0.05

### Multiple linear regression analysis of fetal abdominal overgrowth ratios

In multiple linear regression analyses, maternal age and HbA1c but not BMI at the 50-g GCT exhibited significant positive associations with fetal abdominal overgrowth ratios (Table 5).

**Table 5:** Multiple linear regression analysis of fetal abdominal overgrowth ratios.

Outcome	Variables	Parameter Estimate	Standard Error	Pr > ltl
GA-AC/GA-GCT	Age	0.0019	0.0006	0.0018
	BMI-50-g GCT	0.0001	0.0007	0.8659
	HbA1c	0.0183	0.0071	0.0104
	HOMA-β	-0.0000	0.0000	0.1701
GA-AC/GA-BPD	Age	0.0019	0.0006	0.0043
	BMI-50-g GCT	0.0005	0.0008	0.4888
	HbA1c	0.0134	0.0075	0.0756
	HOMA-β	-0.0000	0.0000	0.4713
GA-AC/GA-FL	Age	0.0018	0.0006	0.0052
	BMI-50-g GCT	0.0000	0.0000	0.9043
	HbA1c	0.0166	0.0073	0.0236
	HOMA-β	-0.0000	0.0000	0.1370

GA-AC, estimated gestational age by abdominal circumference; GA-GCT, actual gestational age by last menstruation period on the 50-g GCT (glucose challenge test) and fetal biometry; BMI-50-g GCT, BMI measured on the 50-g GCT; HbA1c, glycated hemoglobin; HOMA-β, homeostatic model assessment for insulin secretion; GA-BPD, estimated gestational age by biparietal diameter; GA-FL, estimated gestational age by femur length

## Discussion

The issue of universal versus selective screening of GDM still remains unsolved. In 2010, the IADPSG proposed universal screening with one-step 75-g OGTT for the diagnosis of hyperglycemia in pregnancy [12] based on the results of the HAPO study which decided the diagnostic glucose threshold upon the risk for neonatal obesity [13] instead of the risk for maternal progression to DM postpartum [31]. From countries adopting selective screening based on their known risk factors, such as maternal age, various levels of pre-pregnancy BMI, macrosomia ≥4.5kg, previous GDM, and family history of type 2 DM, many researchers have reported that the prevalence of GDM was at least tripled by adopting the IADPSG guidelines and that 15% - 50% of GDM patients who were missed by selective screening exhibited a higher risk for adverse perinatal outcome compared with the NGT subjects [32–36]. Several reports have revealed that this universal screening with stringent diagnostic criteria increased the prevalence of GDM without a significant reduction in maternal and neonatal complications [37–39]. Furthermore, this guideline was not considered to be cost-effective [40], so many European countries favoring selective screening, such as the United Kingdom, Ireland, France, German, Italy, Denmark, and Australia, have tried to improve the performance of their selective screening test by combining various risk factors [32–34,39,41–43].

The IADPSG recommendation has been endorsed by the World Health Organization (WHO) [11], American Diabetic Association [44], American Association of Clinical Endocrinologist [45], and International Diabetes Federation. Recently, in Europe, the International Federation of Gynecology and Obstetrics [46] together with the European Board and College of Obstetrics and Gynecology and the European Association of Perinatal Medicine [47] accepted the IADPSG’s one-step universal screening approach considering the high rates of hyperglycemia in pregnancy in most populations and the poor sensitivity of selective screening test in addition to cost-effectiveness regarding the costs for type 2 diabetes developing in the future.

In Korea, since the mid-1990s, all pregnant women have been screened with 50-g GCT at 24–28 weeks of gestation, as recommended by the Third International Workshop-Conference on Gestational Diabetes Mellitus, and they underwent a 3-h 100-g OGTT if the GCT results were ≥140 mg/dl [48,49]. The Korean Diabetes Association (KDA) recommends universal screening using either two-step, a 3-h 100-g OGTT applying Carpenter-Coustan criteria, or one-step, 2-h 75-g OGTT adopting the IADPSG’s glucose threshold to diagnose GDM [15].

Kim *et al*. reported that the IADPSG’s criteria increased the incidence of GDM by approximately threefold and that GDM diagnosed by the IADPSG but not by Carpenter-Coustan criteria had an increased risk of adverse pregnancy outcomes in the Korean population [50]. The KDA supports both the American College of Obstetricians and Gynecologists (ACOG) [51] guidelines and the IADPSG guidelines [12], but most institutes in Korea adopt the ACOG guidelines because the National Health Insurance program providing universal health coverage currently covers only a two-step test.

In our study adopting a two-step, universal screening at 24–28 weeks of gestation, the prevalence of GDM in the young and nonobese group with no risk factor was only 3.2%. But if we screened only the high-risk old and/or obese group, we might miss about 30% of the GDM subjects considering other risk factors, except maternal obesity and old age.

The prevalence of GDM is higher in older (>35 years) [52,53] and obese (BMI > 29 kg/m^2^) pregnant women [52–54]. In the present study, stark differences were seen in the prevalence of GDM: 8.8% and 6.8% in the younger (<35 years) and obese (BMI≥ 25 kg/m^2^) group 2 and in the older and nonobese group 3, respectively, and 22.4% in the older and obese group 4 compared with just 3.2% in the younger and nonobese group 1. The findings of the highest HOMA-IR in group 2 GDM, the lowest HOMA-β in group 3 GDM, and both high HOMA-IR and low HOMA-β in group 4 GDM suggest that the highest prevalence of GDM in the old and obese group 4 is the additive result of increased insulin resistance due to obesity and decreased insulin secretion due to age [55].

Measurement of AC by fetal ultrasound can be used as a reliable marker of fetal adiposity [26]. In the present study, the estimated GAs instead of the percentile values of fetal parts measured *via* ultrasonography at the same time as the screening for GDM were used to observe asymmetric growth of fetal parts. Calculated ratios such as FL/AC, FL/BPD and head circumference/AC are mainly used to detect fetal growth retardation. To detect the relative abdominal overgrowth, we investigated the ratio of the estimated GA of the AC divided by the actual GA determined by the LMP on the 50-g GCT, the GA of BPD, and the GA of FL in the GDM and NGT subjects. Fetal abdominal overgrowth defined with fetal abdominal overgrowth ratios ≥ 90^th^ percentile is more sensitive than the AC ≥ 90^th^ percentile, because about 2 weeks of acceleration of abdominal growth meet the fetal abdominal overgrowth ratio criteria, but more than 3 weeks of acceleration meet the AC criteria during 26 weeks of gestation.

We observed the fetal abdominal overgrowth relative to the growth of BPD and FL in the GDM subjects already existed at the time of the diagnosis of GDM as in previous reports [25,26], but it was observed only in the older and/or obese GDM subgroups. Furthermore, despite the absence of significant differences in the estimated fetal weight among the study groups, the fetal abdominal overgrowth ratios were significantly higher in the old age group 3 and the highest in group 4 compared with those in the NGT group. There might be a nonsignificant tendency for an increase in these ratios observed in the young and obese group 2 because there were only 16 subjects with available fetal biometry data in this group. Also, in multiple linear regression analyses, maternal age was significantly associated with fetal abdominal overgrowth ratios. Collectively, these findings reveal that maternal age as well as obesity have marked impact on fetal abdominal obesity and suggest that, while elderly pregnancy alone already promotes fetal abdominal overgrowth, the co-presence of maternal obesity more aggravates the fetal abdominal obesity observed in elderly pregnant women at the time of GDM diagnosis.

Maternal obesity increases the risk of fetal abdominal obesity and large-for-gestational-age (LGA) birth [25,26,30]. Unlike the report of Sovio *et al*. [26] showing that the risk of an AC > 90th percentile at 28 weeks of gestation is 2.04 when the fetus is exposed to GDM but 4.52 when the fetus is exposed to GDM with obesity after adjustment for maternal age, we could not find such a marked increase in the risk of fetal abdominal obesity due to the addition of maternal obesity and GDM. In our results, when the fetus was exposed to GDM alone and GDM with obesity, the risks of fetal abdominal overgrowth ratio > 90th percentile (GA-AC/GA-GCT > 1.08) at 26 weeks of gestation were 1.11 and 3.45 (statistically insignificant OR) in the younger group and 2.63 and 3.39 in the older group, respectively. These findings could be explained by differences in ethnicity, the diagnostic criteria of GDM, and the definition of obesity.

There is a close link between maternal hyperglycemia and neonatal adiposity [56,57]. HbA1c helps in screening for GDM [58] and predicting adverse pregnancy events [59,60], even though glucose measurement is reported to be more strongly associated with adverse pregnancy outcomes [57]. In the present study, FPG on 100-g OGTT and HbA1c levels exhibited significant positive correlation with fetal abdominal obesity ratios and HbA1c exhibited higher correlation coefficient than FPG. Multivariate analyses revealed an increased risk of fetal abdominal overgrowth with HbA1c level > 37.7 mmol/mol (5.6%) and significant association of HbA1c with fetal abdominal overgrowth.

However, in the subgroup analysis, older and obese group 4 GDM with FPG of 5.3 mmol/L and HbA1c of 37.7 mmol/mol (5.6%) exhibited highest fetal abdominal overgrowth ratios and increased risk for fetal abdominal overgrowth in comparison with the NGT subjects. But young and obese group 2 GDM did not show a significant difference compared with the NGT group despite the higher FPG and HbA1c. Moreover, significant increases in fetal abdominal overgrowth ratios and risk for fetal abdominal overgrowth were observed in the older and nonobese group 3 GDM with FPG of 4.9 mmol/L and HbA1c of 33.3 mmol/mol (5.2%), but not in younger and nonobese group 1 GDM with similar levels of FPG and HbA1c. HOMA-IR and HOMA-β were comparable in each pair of nonobese groups 1 and 3 subjects and obese groups 2 and 4 subjects, even though they were higher in the obese pair and decreased with age in both pairs. In addition, the fasting plasma insulin levels, which were higher in the obese pair, decreased with age in both paired groups (S2 Fig). Fetal abdominal overgrowth observed in the old and nonobese but not in the young and nonobese GDM subgroup despite comparable glucose and HbA1c levels suggests that decreased maternal insulin secretory capacity in elderly GDM that existed before pregnancy. Decreased acute phase insulin responses to glucose and transient postprandial hyperglycemia which is not captured by HbA1c must have resulted in fetal hyperinsulinemia and ensuing fetal adiposity during early pregnancy. Exaggerated glucose steal by an early established hyperinsulinemic fetus [61] might also explain the lower fasting glucose level in the older and nonobese GDM group compared with the younger GDM group. Taken together, absolute glucose and HbA1c levels alone cannot adequately predict fetal abdominal overgrowth without simultaneous evaluation of maternal insulin secretory capacity and insulin resistance.

Screening for GDM at 24–28 weeks of gestation is currently the commonly used approach based on the evidence that fetal abdominal obesity primarily occurs during late pregnancy [1,62]. We and other researchers observed fetal abdominal overgrowth beginning early in pregnancy [18,22]. Despite normalized birth weight, GDM management did not normalize excess fetal adiposity [22]. Moreover, significantly accelerated fetal abdominal growth observed in groups 3 and 4 GDM subjects indicates that glycemic control during the mid-to-late pregnancy after diagnosis of GDM at 24–28 weeks of gestation is inadequate to normalize fetal hyperinsulinemia induced early in pregnancy [16,17] and, thus, cannot attenuate the already triggered fetal adiposity observed in older and particularly obese pregnant women.

The critical timing for the early screening diagnosis and management of GDM remains unclear. The IADPSG recommends screening in all or only high-risk women to detect overt diabetes and early GDM at the initial perinatal visit [12], and this recommendation is accepted by the WHO [11] and the Endocrine Society [6], but early GDM, which is a lesser degree of hyperglycemia detected during the initial prenatal visit, is highly debatable with regard to the screening time, diagnostic criteria, intensity of treatment, and risk factors [63–65]. The KDA also recommends screening all women during the initial prenatal visit to identify overt diabetes. But there is no established recommendation for women with glucose levels in the pre-diabetic range in early pregnancy [15].

Fetal hyperinsulinemia and the ensuing fetal fat deposition are observed as early as 14 weeks of gestation [66]. Blood glucose levels under the diabetic range at 9–10 weeks of gestation [67] and HbA1c level during the first trimester [68] have been reported to show a positive correlation with the prevalence of LGA birth. Early screening and treatment of GDM in high-risk women improves pregnancy outcomes [69]. Despite early screening, diagnosis, and current best practice management of GDM, poorer pregnancy outcomes, including macrosomia are observed in early-onset GDM compared with late-onset GDM [7,70].

According to our results, the routine diagnosis of GDM at 24–28 weeks of gestation is too late to prevent fetal abdominal obesity in old and/or obese GDM subjects. So, all women should be screened during the initial prenatal visit, earlier than 12 weeks of gestation in addition to universal screening at 24–28 week of gestation. If a lesser degree of hyperglycemia than overt DM is detected in women, they should be diagnosed as having early GDM with lower glucose threshold than impaired fasting glucose in the nonpregnant population because of emesis and increased glucose consumption due to fetal organogenesis in early pregnancy. The glucose threshold which is below the diagnostic range of diabetes but needs intervention in early pregnancy should be decided through well-designed prospective studies.

The limitations of the present study include the single-center, retrospective, and uncontrolled observational study design. Although maternal age and obesity were stratified, other unknown confounders might not have been controlled for in this retrospective study. We excluded subjects who were diagnosed with impaired fasting glucose or impaired glucose tolerance before pregnancy and were diagnosed with pregnancy-induced hypertension before the 50-g GCT, who were screened early for any reasons such as glycosuria or past history of macrosomia and exhibited abnormal results, and who were ordered but without the results of 50-g GCT. However, we did not screen early for all known risk factors. Above all, interobserver variability on the assessment *via* the ultrasonography was not evaluated because this study was retrospective. But there were no differences in the ultrasound scanners used, and all pregnant women scanned were randomly assigned to the three observers. We could not obtain insulin data to calculate acute-phase insulin secretion. However, the strengths of this study include a relatively large sample size with the same ethnicity and clinical management of all subjects according to the same protocol during the study period.

In summary, the prevalence of GDM is higher in older or obese pregnant women, and even higher in older and obese women, compared with younger and nonobese women. Fetal abdominal obesity is already evident at the time of GDM diagnosis at 24–28 weeks of gestation in older and nonobese/obese women, but not in younger and nonobese women. In addition, the risk for fetal abdominal obesity is significantly increased with maternal obesity, older age, and high HbA1c. Based on these findings, it can be suggested that, even though the current GDM screening strategy at 24–28 weeks of gestation might be effective for younger and nonobese women, GDM screening diagnosis and the best appropriate management starting from early pregnancy might be necessary for high-risk older and particularly obese GDM mothers to prevent fetal abdominal obesity.

## Supporting information

S1 Table Prevalence of GDM according to maternal age and BMI. (PDF)

S2 Table Factors related to fetal abdominal overgrowth in all of the subjects. (PDF)

S3 Table Factors related to fetal abdominal overgrowth in the GDM subjects. (PDF)

S1 Fig Measurements of fetal biparietal diameter, femur length, and abdominal circumference by ultrasonography and conversion to estimated gestational age. (TIF)

S2 Fig Insulin resistance and insulin secretion of the GDM and NGT subjects in each group. (TIF)
