# Supplementary material for: Gestational diabetes mellitus diagnosed at 24 to 28 weeks of gestation in older and obese Women: Is it too late?
Source: PLoS One. 2019 Dec 16;14(12):e0225955. doi: 10.1371/journal.pone.0225955 (PMC6913988; doi:10.1371/journal.pone.0225955)
Supplement: S1 Table — (PDF) [file pone.0225955.s001.pdf]

Table S1. Prevalence of GDM according to maternal age and BMI

	Age <35 years<br>(n = 4927)	Age ≥35 years<br>(n = 2642)	P-value	Total
BMI < 5 kg/m^2<br>(n = 7079)	3.2 %<br>(147/4665)	6.8 %<br>(163/2414)	< 0.0001	4.4 %<br>(310/7079)
BMI ≥ 25 kg/m^2<br>(n = 490)	8.8 %<br>(23/262)	22.4 %*<br>(51/228)	< 0.0001	15.1 %<br>(74/490)
P-value	<0.0001	<0.0001		
Total<br>(n = 7569)	3.5 %<br>(170/4927)	8.1 %<br>(214/2642)		5.1 %<br>(384/7569)

GDM, gestational diabetes mellitus; BMI, body mass index

*P-value <0.0001 compared with Group 1 (age <35 years and BMI <25kg/m^2)
