# Supplementary material for: Gestational diabetes mellitus diagnosed at 24 to 28 weeks of gestation in older and obese Women: Is it too late?
Source: PLoS One. 2019 Dec 16;14(12):e0225955. doi: 10.1371/journal.pone.0225955 (PMC6913988; doi:10.1371/journal.pone.0225955)
Supplement: S2 Table — (PDF) [file pone.0225955.s002.pdf]

Table S2. Factors related to fetal abdominal overgrowth in all subjects

Fetal Abdominal Overgrowth Ratio	Variables	Correlation coefficient	p-value
GA-AC/GA-GCT	Age	0.1140	<0.0001
	Pre-pregnancy BMI	0.0944	<0.0001
	BMI-50-g GCT	0.1320	<0.0001
	Weight gain	0.0943	<0.0001
	FPG	0.1477	<0.0001
	HbA1c	0.1619	<0.0001
	HOMA-β	- 0.1148	0.0209
	HOMA-IR	0.00210	0.6575
GA-AC/GA-FL	Age	0.0751	<0.0001
	Pre-pregnancy BMI	0.0681	<0.0001
	BMI-50-g GCT	0.0970	<0.0001
	FPG	0.1358	<0.0001
	Weight gain	0.0911	<0.0001
	HbA1c	0.1452	<0.0001
	HOMA-β	- 0.1154	0.0203
	HOMA-IR	0.01785	0.7206
GA-AC/GA-BPD	Age	0.0912	<.0001
	Pre-pregnancy BMI	0.0944	<.0001

	BMI-50-g GCT	0.0981	<.0001
	Weight gain	0.0086	0.5242
	FPG	0.1342	<0.0001
	HbA1c	0.1537	<.0001
	HOMA-β	-0.0572	0.2530
	HOMA-IR	0.0115	0.8206

GA-AC, estimated gestational age by abdominal circumference; GA-GCT, actual gestational age by last menstruation period (LMP) at 50-g GCT (glucose challenge test) and fetal biometry; BMI-50-g GCT, BMI measured at 50-g GCT; FPG, fasting plasma glucose on 100g OGTT; HbA1c, glycated hemoglobin; HOMA-β, homeostatic model assessment for insulin secretion; HOMA-IR, homeostatic model assessment for insulin resistance; GA-FL, estimated gestational age by femur length ; GA-BPD, estimated gestational age by biparietal diameter;
