# Supplementary material for: Gestational diabetes mellitus diagnosed at 24 to 28 weeks of gestation in older and obese Women: Is it too late?
Source: PLoS One. 2019 Dec 16;14(12):e0225955. doi: 10.1371/journal.pone.0225955 (PMC6913988; doi:10.1371/journal.pone.0225955)
Supplement: S3 Table — (PDF) [file pone.0225955.s003.pdf]

Table S3. Factors related to fetal abdominal overgrowth in GDM subjects

Fetal Abdominal Overgrowth Index	Variables	Correlation Coefficient	P-value
GA-AC/GA-GCT	Age	0.1724	0.0032
	Pre-pregnancy BMI	0.0859	0.1472
	BMI-50-g GCT	0.1480	0.0122
	Weight gain	0.1612	0.0063
	FPG	0.1905	0.0012
	HbA1c	0.2211	0.0002
	HOMA-β	- 0.1040	0.0904
	HOMA-IR	0.0113	0.8544
GA-AC/GA-FL	Age	0.1443	0.0137
	Pre-pregnancy BMI	0.0570	0.3371
	BMI-50-g GCT	0.1180	0.0461
	Weight gain	0.1427	0.0157
	FPG	0.1810	0.0022
	HbA1c	0.2208	0.0002
	HOMA-β	- 0.1134	0.0649
	HOMA-IR	0.0032	0.9583
GA-AC/GA-BPD	Age	0.1477	0.0188
	Pre-pregnancy BMI	0.0952	<.0001

	BMI-50-g GCT	0.0987	<.0001
	Weight gain	0.0085	0.5262
	FPG	0.1750	0.0032
	HbA1c	0.151	<.0001
	HOMA-β	-0.0373	0.4590
	HOMA-IR	0.0042	0.8583

GA-AC, estimated gestational age by abdominal circumference; GA-GCT, actual gestational age by last menstruation period (LMP) at 50-g GCT (glucose challenge test) and fetal biometry; BMI-50-g GCT, BMI measured at 50-g GCT; FPG, fasting plasma glucose on 100g OGTT; HbA1c, glycated hemoglobin; HOMA-β, homeostatic model assessment for insulin secretion; HOMA-IR, homeostatic model assessment for insulin resistance; GA-FL, estimated gestational age by femur length ; GA-BPD, estimated gestational age by biparietal diameter;
