# Supplementary figures and images for: Gestational diabetes mellitus diagnosed at 24 to 28 weeks of gestation in older and obese Women: Is it too late?
Source: PLoS One. 2019 Dec 16;14(12):e0225955. doi: 10.1371/journal.pone.0225955 (PMC6913988; doi:10.1371/journal.pone.0225955)

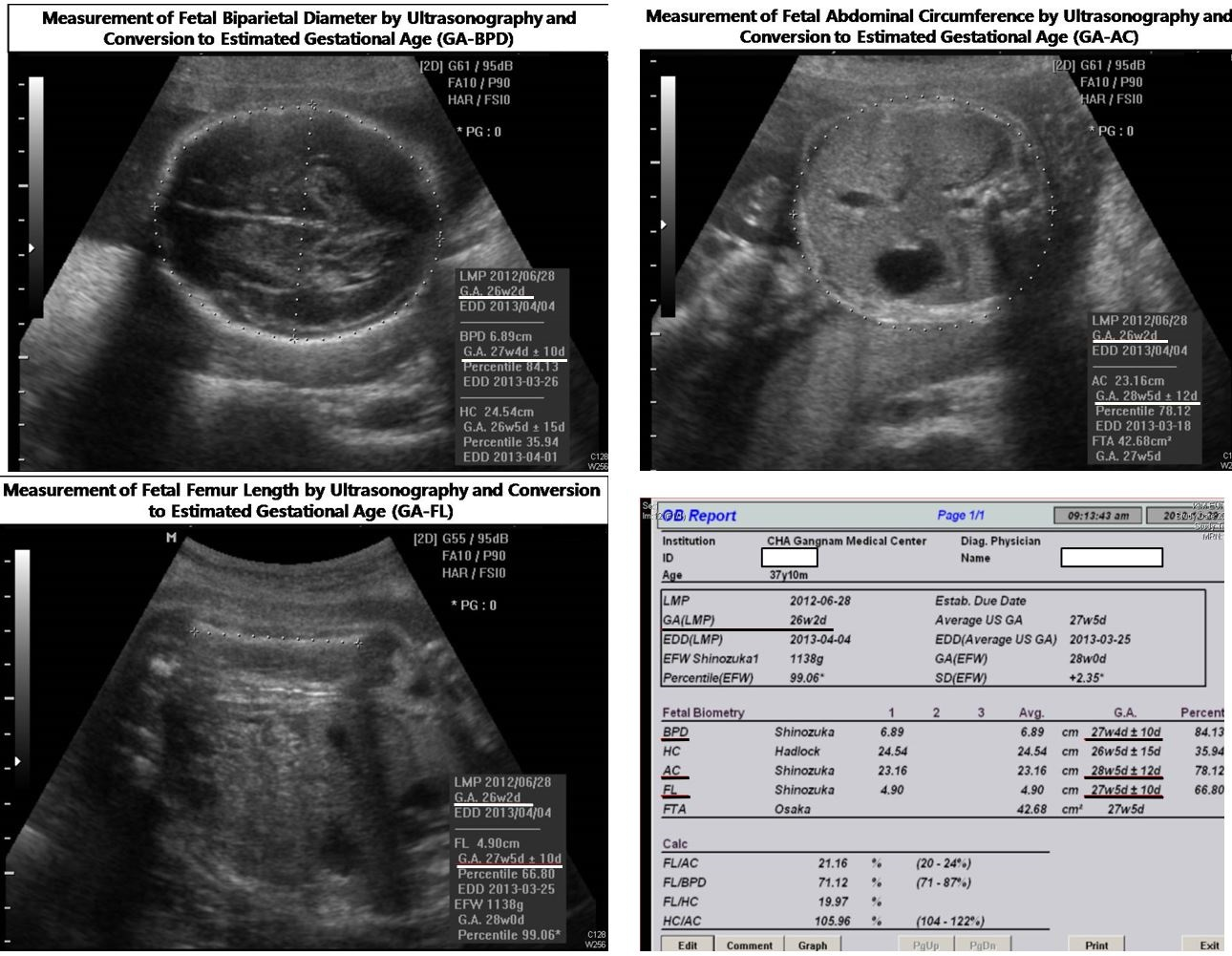

Supplement: S1 Fig — (TIF) [file pone.0225955.s004.tif]

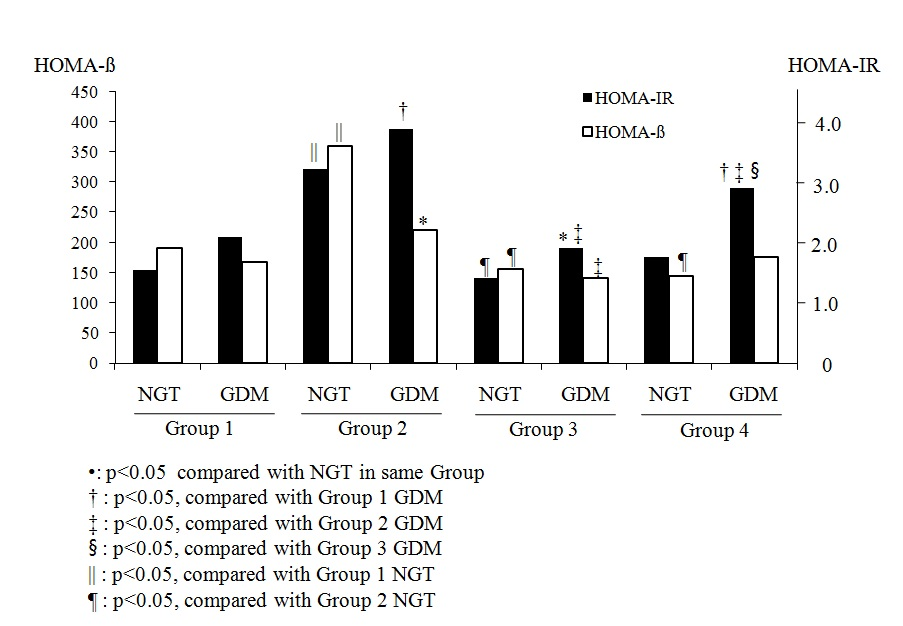

Supplement: S2 Fig — (TIF) [file pone.0225955.s005.tif]
